# *Cryptococcus neoformans* capsule regrowth experiments reveal dynamics of enlargement and architecture

**DOI:** 10.1016/j.jbc.2022.101769

**Published:** 2022-02-24

**Authors:** Maggie P. Wear, Ella Jacobs, Siqing Wang, Scott A. McConnell, Anthony Bowen, Camilla Strother, Radames J.B. Cordero, Conor J. Crawford, Arturo Casadevall

**Affiliations:** W. Harry Feinstone Department of Molecular Microbiology and Immunology, Johns Hopkins Bloomberg School of Public Health, Baltimore, Maryland, USA

**Keywords:** sonication, polysaccharide, capsule, cryptococcus, Glucanex, BMDM, bone marrow–derived macrophage, CPS, capsular polysaccharide, CTAB, cetyl trimethylammonium bromide, DMSO, dimethyl sulfoxide, EPS, exopolysaccharide, GXM, glucuronoxylomannan, IF, immunofluorescence, PSA, phenol sulfuric acid, SRG, structural reporter group, QCA, quantitative capsule analysis

## Abstract

The polysaccharide capsule of fungal pathogen *Cryptococcus neoformans* is a critical virulence factor that has historically evaded complete characterization. Cryptococcal polysaccharides are known to either remain attached to the cell as capsular polysaccharides (CPSs) or to be shed into the extracellular space as exopolysaccharides (EPSs). While many studies have examined the properties of EPS, far less is known about CPS. In this work, we detail the development of new physical and enzymatic methods for the isolation of CPS which can be used to explore the architecture of the capsule and isolated capsular material. We show that sonication or Glucanex enzyme cocktail digestion yields soluble CPS preparations, while use of a French pressure cell press or Glucanex digestion followed by cell disruption removed the capsule and produced cell wall–associated polysaccharide aggregates that we call “capsule ghosts”, implying an inherent organization that allows the CPS to exist independent of the cell wall surface. Since sonication and Glucanex digestion were noncytotoxic, it was also possible to observe the cryptococcal cells rebuilding their capsule, revealing the presence of reducing end glycans throughout the capsule. Finally, analysis of dimethyl sulfoxide-extracted and sonicated CPS preparations revealed the conservation of previously identified glucuronoxylomannan motifs only in the sonicated CPS. Together, these observations provide new insights into capsule architecture and synthesis, consistent with a model in which the capsule is assembled from the cell wall outward using smaller polymers, which are then compiled into larger ones.

The survival of *Cryptococcus* spp*.* in nature requires the yeast to defend itself against environmental stresses and phagocytic predators. Factors that afford this protection are hypothesized to function as virulence factors in the mammalian host (1, 2, 3). One such factor is the polysaccharide capsule of *Cryptococcus neoformans,* which protects the microbe from environmental desiccation (4) and amoeba predation (1). The capsule is comprised of at least two polysaccharides that have immunomodulatory and immunosuppressive activity: glucuronoxylomannan (GXM) and galactoxylomannan as well as mannoproteins at low abundance (5), though both galactoxylomannan and mannoproteins are hypothesized to only be secreted, not maintained in the capsule (6). Capsule size also plays a role in virulence and immune evasion (7). Larger capsule sizes are associated with more severe clinical outcomes as well a reduction in phagocytosis (8, 9). Establishing the essential nature of the capsule in pathogenicity, acapsular mutants exhibit a striking loss of virulence (10, 11).

Cryptococcal polysaccharides are either attached to the cell as capsular polysaccharide (CPS) or are shed into the surroundings in the form of exopolysaccharide (EPS). Both CPS and EPS contribute to the immunosuppressive activity ascribed to the capsule, yet the two are antigenically and chemically distinct (12, 13, 14). A great deal is known about the properties of EPS due to well-established isolation protocols and ease of isolation. Until recently, most studies of EPS relied on cetyl trimethylammonium bromide (CTAB) extraction (15). CTAB is a detergent that can persist in the polysaccharide sample, hampering purification (12). Additionally, CTAB processing requires dehydration of the polysaccharide molecules, potentially collapsing or otherwise altering polysaccharide structure irreversibly. More recent work shows that EPS isolation can also be achieved by filtration methods that jellify the polysaccharide molecules, largely maintaining their hydrated, native state (12, 16).

In comparison with EPS, there is a relative dearth of literature describing CPS structure and function. The capsule is a highly hydrated structure composed primarily of water (17) presenting a major challenge to CPS isolation, as well its association with the cell wall. The most utilized methods for CPS isolation in the literature are gamma irradiation and dimethyl sulfoxide (DMSO) extraction. Both methods have limitations. DMSO, like CTAB, persists in the sample after isolation, and both the mechanism of action for capsule removal and potential structural alterations of the higher order of CPS are unknown. Gamma irradiation, which is thought to break glycosidic and other bonds through the radiolysis of water (5, 18, 19, 20), could result in the indiscriminate breakage of bonds, including the formation of open ring structures in the isolated polysaccharides (21, 22).

To address the need for new methodologies in the isolation of CPS, we have explored physical and enzymatic methods of disrupting the linkage between the cell and capsule. This work describes three new methods of capsule disruption that add to the arsenal of capsular probing tools: ultrasonication, French press, and Glucanex digestion. Ultrasonication is a popular method used to perturb cells for lysis (23, 24). Ultrasonic sound waves (above 16 kHz) cause microscopic bubbles to form in solution. Collapse cavitation occurs when the bubbles implode resulting in high pressures and temperatures that can cause mechanical damage to surrounding materials (25). Stable cavitation is the consequence of oscillations in bubble size, and this results in microstreaming (rapid flow of medium around the bubble), which produces sheer forces strong enough to damage macromolecules (25, 26). The French pressure cell press disrupts the *C. neoformans* capsule with a piston that exerts hydraulic pressure on the cell culture solution, forcing it through a narrow valve opening (27, 28). The principle of French press extorts fluid dynamics but with the application of high-pressure forces added to disrupt cell walls. Glucanex, or lysing enzymes from *Trichoderma harzianum*, contains β-1,3-glucanase, cellulase, protease, chitinase, and the more recently discovered ɑ-1,3-endo-glucanase (29). This enzyme cocktail has been utilized for the production of *C. neoformans* protoplasts, thus removing the cell wall, which in this work is shown to also include the capsule (30). In this work, we use these new methods to gain new insights into capsule architecture and the composite polysaccharides.

## Results

### Characterization of capsule isolation by sonication, Glucanex, and DMSO

**Figure 1:**
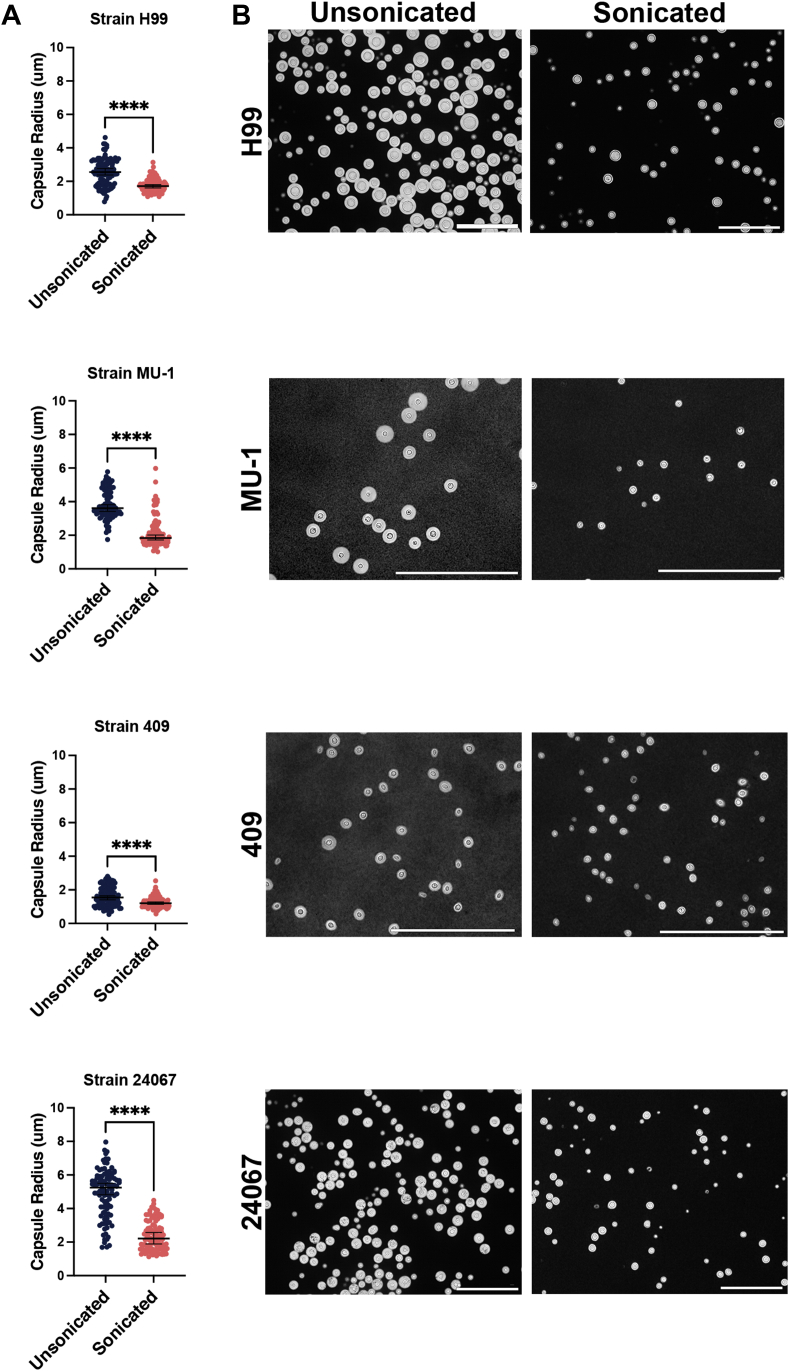
**Effect of sonication on the capsule radius of *C. neoformans* serotype A and D strains.***A*, quantitative capsule measurement before and after sonication in serotype A strains H99 and single motif expressing MU-1, serotype B strain 409, and serotype D single motif expressing strain 24,067. n = 100 cells were measured for each strain in each condition. *B*, india ink microscopy images of strains before (*left*) and after (*right*) sonication. Scale bars represent 100 μm. Unpaired students *t* test were performed on all strains to compare sonicated and unsonicated cells. n = 100 for each strain and condition. ∗∗∗∗ represents *p* value <0.0001.

**Figure 2:**
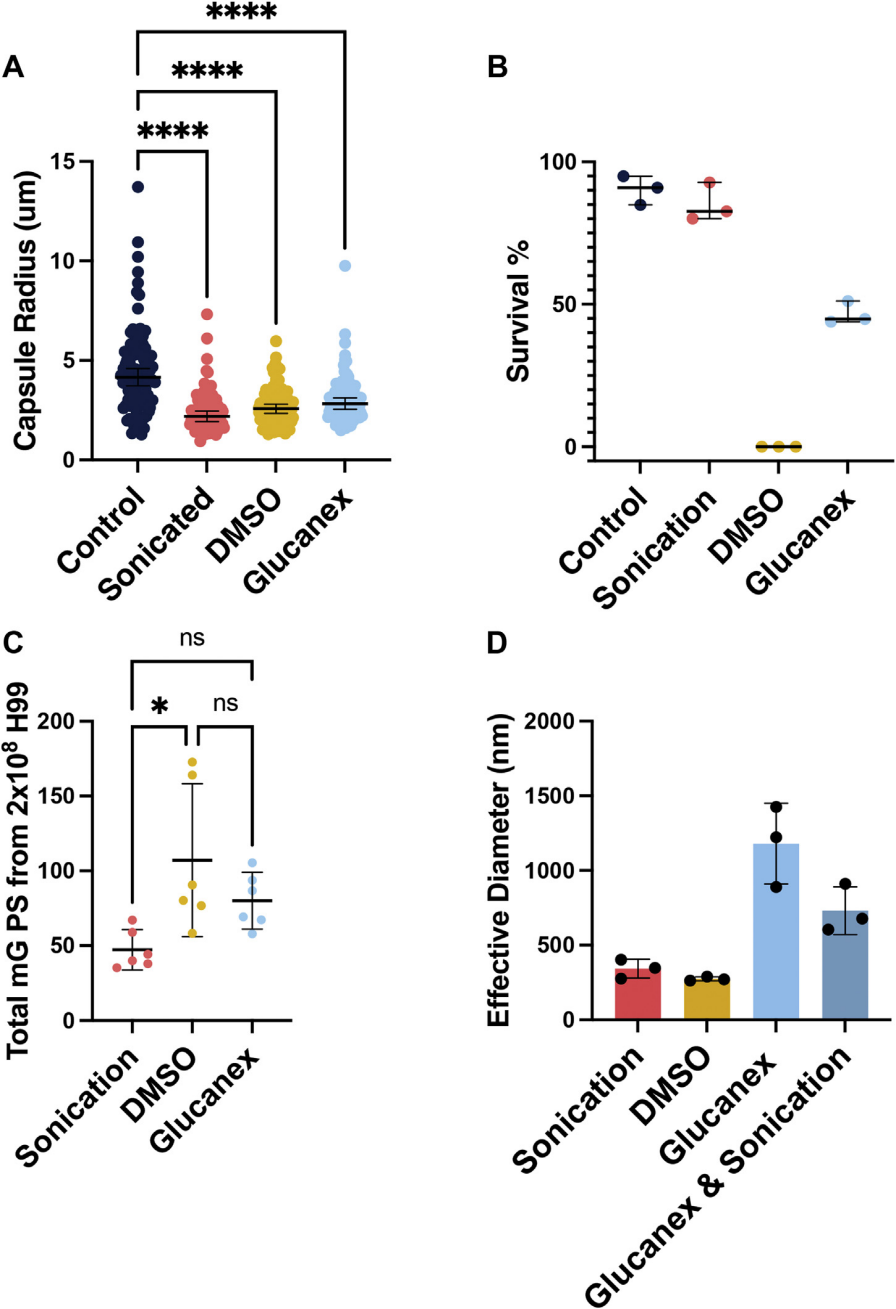
**Comparison of capsular removal methods to impact radius, cell viability, and released polysaccharide.***A*, quantitative measure of removal of capsule by sonication, DMSO, or Glucanex treatment. Capsule radius was measured for 100 cells for each treatment. Capsule radius was compared between strains using a one-way ANOVA. ∗∗∗∗ indicates a *p* value <0.0001. *B*, methylene blue survival of *C. neoformans* cells before (control) and after CPS removal. Values represent three biological replicates each. *C*, total removed polysaccharide estimated by sandwich ELISA from 2 × 10^8^ H99 cells. Values represent three biological replicates with two technical replicates each. Amount of removed polysaccharide was compared using an ANOVA. ∗represents a *p* value <0.05. *D*, effective particle diameter estimated by dynamic light scattering analysis of three independent CPS isolations. Bars represent the mean of three biological replicates with standard deviation shown. CPS, capsular polysaccharide; DMSO, dimethyl sulfoxide.

We evaluated the outcome of treating *C. neoformans* cells by sonication and with Glucanex, compared to those treated with DMSO. Sonication resulted in a statistically significant reduction in capsule size in three *C. neoformans* strains, including those of serotype A, B, and D. (Fig. 1*A*). The starting capsule and cell body size varied with the strain, yet sonication efficiently removed material from all strains (Figs. 1*B* and S1), leaving a median capsule radius after treatment of approximately 2 μm (Figs. 1*A* and 2*A*). Glucanex is usually used in the cryptococcal field to generate spheroplasts as a prelude to lysing cells. Here, we note that in removing the H99 cell wall, it also removes the polysaccharide capsule. For each of the three methods, we measured changes to H99 cells: (i) reduction in capsule radius, (ii) cell survival, (iii) freed polysaccharide as measured by capture ELISA and phenol sulfuric acid (PSA) assays, and (iv) size of polymers in isolated CPS. Cells were analyzed for capsule-size reduction using quantitative capsule analysis (QCA), a computer program developed in our laboratory that scans microscopic images to provide capsule dimension information (31). All three capsule removal methods produced a significant reduction in capsule size (Fig. 2*A*), but DMSO treatment completely killed the cells while both sonication and Glucanex digestion were significantly less toxic, reducing viability to ∼90% and ∼60%, respectively (Fig. 2*B*).

ELISA assays estimated a total polysaccharide yield from 2 × 10^8^ cells of 99 mg for DMSO, 78 mg for Glucanex treatment, and 46 mg for sonication (Fig. 2*C*). These polysaccharide yields are consistent with analysis of wet cell pellets before and after sonication, with the same number of cells yielding a total mass of 138 mg, which decreased to 98 mg after sonication, indicating that 44 mg of polysaccharide was released, a value that is close to that measured by ELISA. While capsule diameter is weakly negatively correlated with ELISA-measured supernatant GXM, PSA measurements of the same supernatant show less correlation with capsule diameter. Thus, capsule diameter measurements and GXM quantification by ELISA are correlated, but PSA measurements are uncoupled. Since antibody-based detection methods which rely on immunocomplexes between antigen and specific antibodies for capture and detection and are routinely used in clinical and research settings, we utilized the capture ELISA data for quantification.

Measurement of isolated CPS particle size showed high variance between biological replicates, but average effective diameter indicates that Glucanex digestion yielded the largest polymers while DMSO and sonication yield smaller, similarly sized particles (Fig. 2*D*). Additionally, CPS preparations by sonication or Glucanex treatment yielded a concentrated sample of CPS from 2 × 10^8^ cells in just 2 ml while the same number of cells results in 120 ml of preparation by DMSO. The results of one biological replicate examining isolated polysaccharides from single motif expressing strains Mu-1 and 409 show similar sizes between DMSO and sonication removed particles. There was an average effective diameter of 3224 nm with a standard deviation of 733.1 nm for four technical replicates of Mu-1 sonicated CPS and an effective diameter of 1501 nm for one replicate of Mu-1 DMSO extracted CPS. The size of 409 CPS more closely resembled that of H99 with an average of 540.8 nm with a standard deviation of 145.9 nm for 409 sonicated with four technical replicates CPS and 483.2 nm for one replicate of 409 DMSO-extracted CPS (Fig. S2).

We also examined the structure of these sample’s sonication and DMSO CPS preparations.

**Figure 3:**
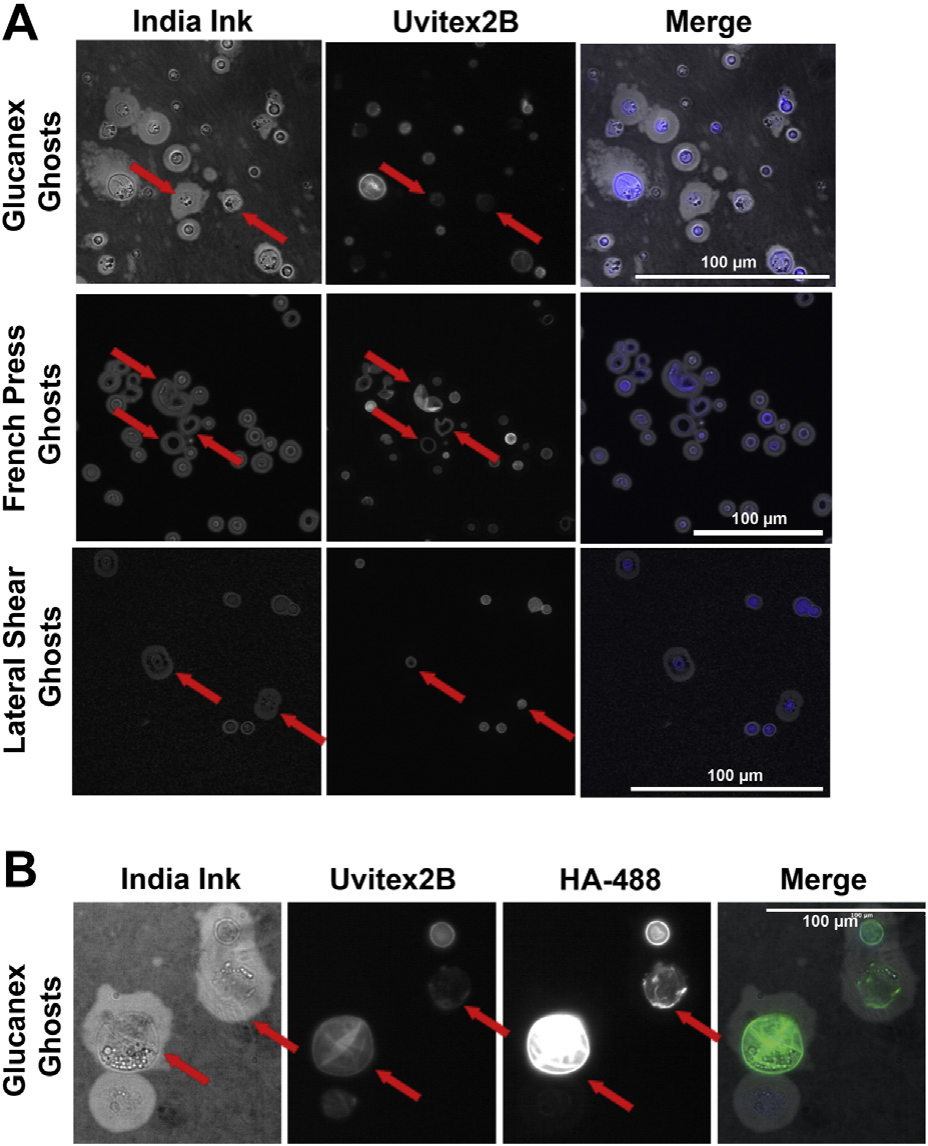
**Glucanex, French press, and lateral shearing treatment produce capsule ghosts**. *A*, india Ink microscopy of capsule ghosts resulting from Glucanex digestion (*top*), French press (*middle*), or lateral shearing (*bottom*) show that capsule ghosts contain cell wall (as stained by Uvitex 2b) but are abnormal in shape, cracked open, blebbing, with off-center cells. Scale bars represent 100 μm. *B*, india Ink microscopy with a polysaccharide reducing end probe (HA-488) and cell wall staining (Uvitex-2b) showing that reducing ends are found at the cell wall even after the cell has been lost from ghosts. Scale bars represent 100 μm*. Red arrows* indicate capsule ghosts.

To include a published standard, we prepared EPS by the previously described CTAB method (32) for two single-motif expressing strains: serotype A strain Mu-1 which expresses the M2 motif and serotype B strain 409 which expresses the M3 motif (Fig. S3*A*). H99 was not used for this analysis because the GXM motifs have not been described. We also prepared CPS from these strains by sonication and DMSO extraction. To overcome the significant challenge of polysaccharide dilution in the DMSO sample, both of DMSO CPS preparations were concentrated by lyophilization from 30 ml to 500 μl for NMR analysis. Analysis of the reported GXM structural reporter group (SRG) region of the 1D spectra (5.0–5.4) shows the signature three peaks in the CTAB EPS spectra for both strains (Fig. S3*A*) (15). These signature peaks are also observed in the sonicated CPS samples (Fig. 3*B*), though with less intensity and requiring more iterations of deconvolution (500 *versus* standard 100) which is likely due to the larger size of capsular fragments compared to the shed EPS fragments. We would note an additional peak was observed in the sonicated Mu-1 CPS sample (highlighted in blue) and is attributed to incomplete de-O-acetylation (acetylated samples contain only this peak) which may also contribute to the additional peaks found in the DMSO CPS samples for both strains. Unfortunately, the SRG peak sets for GXM motifs were not observed in the DMSO CPS sample (Fig. S3*C*). We would note that previous work has reported SRG GXM motif peak sets in DMSO preparations of CPS (12), but without spectra for comparison. Additionally, both the DMSO and sonicated CPS preparations are large in size which results in long T1 relaxation times and line broadening resulting in poor signal to noise.

### Glucanex and French press treatment produce capsule ghosts

**Figure 4:**
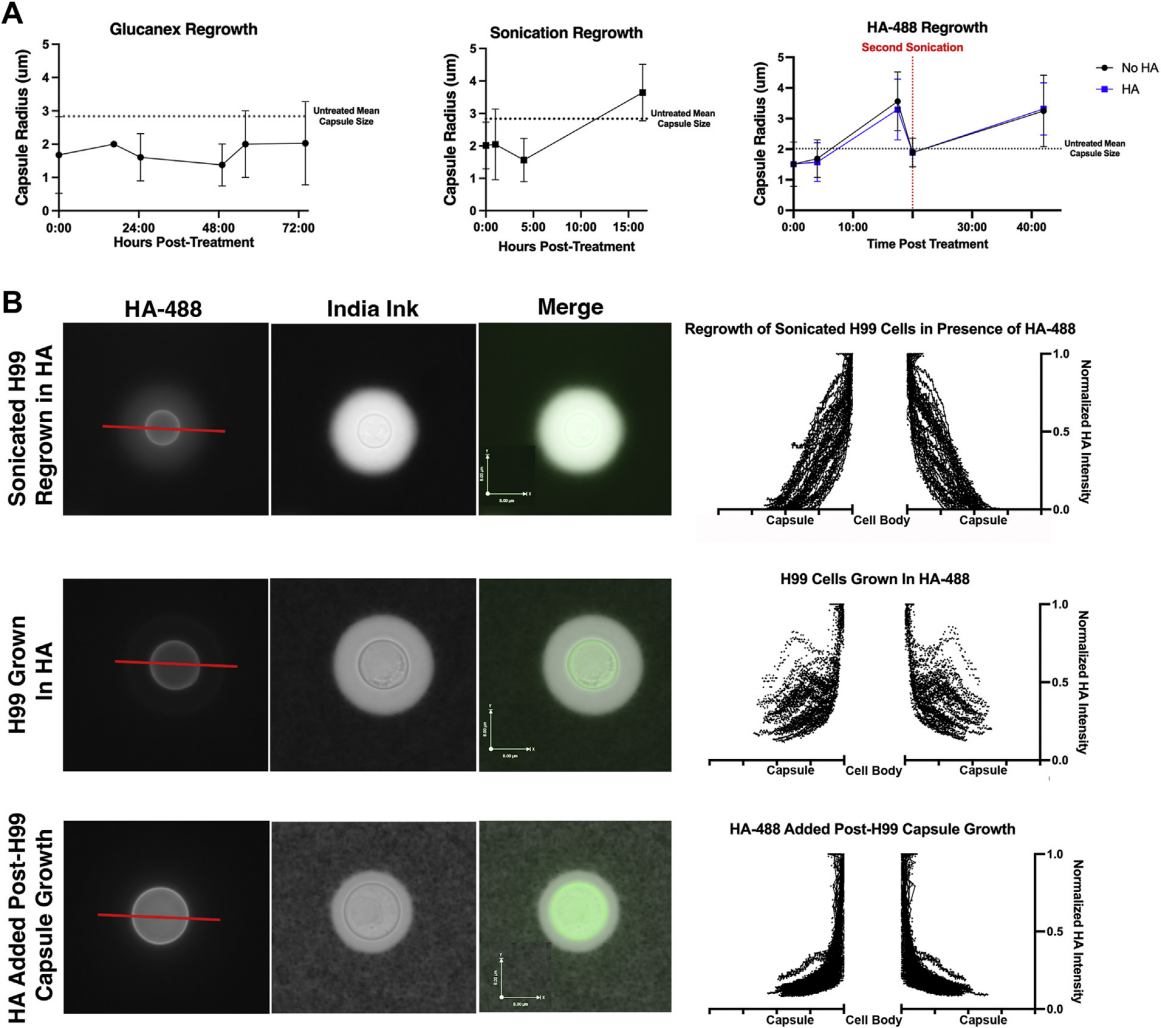
**Examination of capsule regrowth after sonication and Glucanex digestion.** Quantitative capsule radius measurements were taken for all experiments, before (untreated mean), after (0 m), and at specified time intervals. *A*, capsule growth was measured over 76 h post Glucanex digestion (*left*), over 17 h postsonication (*middle*), or after two sonication events in the presence or absence of HA-488 reducing end probe (*right*). For all experiments, before (untreated mean), after (0 m), and at time intervals after cells were returned to minimal medial culture, and a 1 ml aliquot was removed, washed, and imaged with india ink. At each time point, 100 cells were evaluated by QCA to determine median capsule size (listed on plot). The HA-488 stained cells show statistically significantly smaller capsules at 17 h (unpaired student’s *t* test, ∗ represents *p*-value <0.05). *B*, immunofluorescence of reducing end staining with HA-488 throughout cryptococcal capsule. Cells were incubated in the presence of HA-488 reducing end stain during *de novo* capsule growth (*left*, *middle*), after sonication (*left*, *top*), or post capsule growth (*left*, *bottom*). Plot profiles on the right show reducing end staining patterns in capsules as determined by pixel intensity. Plot profiles were normalized to peak intensity to graphically display staining throughout the capsule. Scale bars 8 μm. Greater than 30 cells were analyzed for each sample. QCA, quantitative capsule analysis.

During the processing and analysis described above, we observed what appeared to be capsule structures that no longer contained a cell. In an analogy to a technique to produce melanin ghosts (33), we refer to these structures freed of their cells as capsule ghosts (Fig. 3). Capsule ghosts are produced by Glucanex digestion, 500 psi in a French press, or lateral shearing. All three methods result in abnormally shaped capsules, but French press results in cells that are cracked open while Glucanex digestion and lateral shearing—produced by finger-pressing the encapsulated *C. neoformans* cells between a microscope slide and cover slip—results in capsular blebbing and capsules absent a cell but retaining the cell wall (as confirmed by staining with Uvitex2B) (Fig. 3*A*). Further immunofluorescence (IF) analysis of the Glucanex-derived capsule ghosts showed that they did not contain cellular material, as revealed by the absence of DAPI staining, but did contain GXM as evident by reactivity with mAb 18B7 (Fig. S4). The Glucanex-derived capsule ghosts also had polysaccharide reducing ends, as inferred from reactivity of aldehyde groups with a reducing end probe (hydroxylamine-488) (34), in the area where the capsule would have been in contact with the cell wall (Fig. 4*B*). During optimization, it was noted that yeast cell concentration was important for French press. Cellular breakage easily occurred at a concentration of 1 × 10^7^ and a low pressure of 500 psi. We observed that cells at a concentration of 1 × 10^8^ did not show a reduction in capsule size when exposed to the French press at 500 psi for two passes.

### Sonication-treated cells regrow their capsules with reducing ends throughout the capsule

Noting that sonication and Glucanex digestion removed capsules without killing *C. neoformans* provided the first opportunity for studying capsule regrowth. Prior studies of capsule growth were limited to measuring capsule enlargement (32, 35, 36) after placing cells in capsule enlarging conditions, while sonication and Glucanex digestion provide the opportunity to study the repair of these structures after removal of large portions of polysaccharide. We observed that after 17 h, the sonicated cells had completely regrown their capsules (Fig. 4*A*), but Glucanex-treated cells took longer, requiring more than 74 h to regrow their capsules and not reaching the capsule size of the untreated cells (Fig. 4*A*). We also evaluated where reducing end-containing polysaccharides were incorporated into the capsule after sonication by adding the HA-488 reducing end probe to the culture during capsule regrowth. After 18 h, both the sonicated cells alone and with the reducing end probe had regrown their capsules, though those with the HA-488 lagged behind the control cells (Fig. 4*A*). Further, cells exposed to a second round of sonication were able to regrow their capsules again (Fig. 4*A*). In both these circumstances, staining from the reducing end probe was observed throughout the regrown capsule (Fig. 4*B*). To confirm that this staining pattern was not due to disruption of the capsule by sonication, cells were cultured for 3 days in minimal media in the presence of HA-488 with no sonication, and capsule growth was observed (Fig. 4*B*). Staining from the reducing end probe was observed throughout the capsule in cells grown in the presence of HA-488 without sonication. This pattern differs from that of cells stained after growth in minimal media, both confirming our previously reported observation that reducing ends staining is not observed in mature capsules and showing that reducing end staining is observed as during capsule growth (34). Images of cells with no staining were analyzed to confirm capsular staining was not due to background autofluorescence (Fig. S5).

### Effects of sonication on antibody and complement binding and phagocytosis

**Figure 5:**
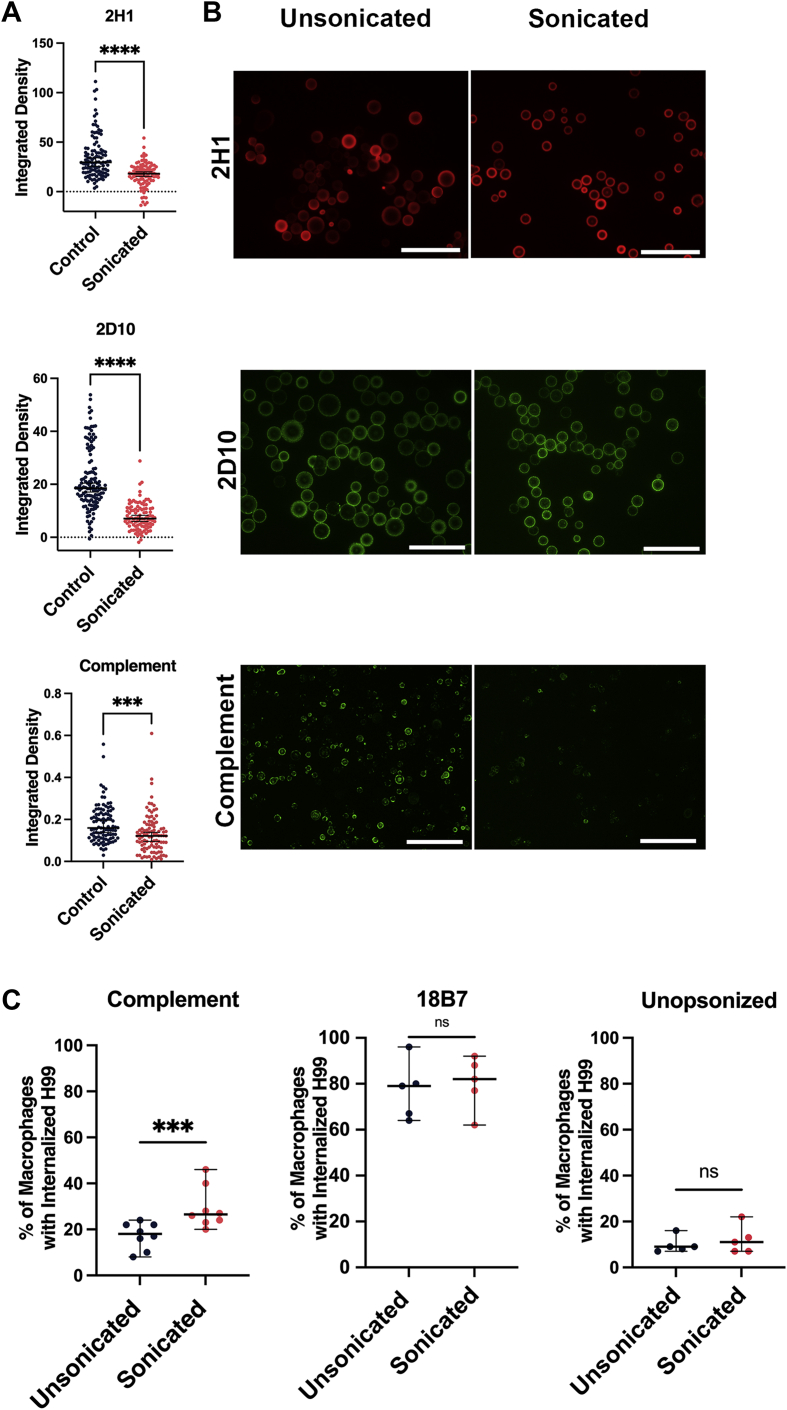
**Effect of sonication on antibody and complement binding to the capsule.***A*, integrated density analysis of immunofluorescence staining of *C. neoformans* cells before and after sonication. An average of 104 cells with a standard deviation of 10.99 were analyzed for each condition. Conditions were compared using an unpaired students *t* test. ∗∗∗ represents a *p* value <0.005, ∗∗∗∗ represents a *p* value <0.0001. *B*, immunofluorescence microscopy images of strains before (*left*) and after (*right*) sonication with anti-GXM IgG 2H1, IgM 2D10, or complement. Scale bars represent 100 μm. *C*, percentage of BMDM cells with internalized cryptococcus *via* complement-mediated (*left*), antibody-mediated (*middle*) mechanisms, and non-opsonized (*right*) H99 before and after sonication. Data represent eight independent experiments for complement opsonization, five for antibody opsonization, and five for each strain’s representative GXM motif. BMDM, bone marrow–derived macrophage; GXM, glucuronoxylomannan.

To assess how the sonication-mediated removal of CPS affected the capsule of *C. neoformans,* we performed IF staining and microscopy with two different mAbs to GXM. While capsule reduction analysis shows that not all the capsule is removed by sonication, IF shows that the staining of mAbs 2D10 (IgM) and 2H1 (IgG) was less intense (Fig. 5*A*). Both antibodies stained the capsule before and after sonication (Fig. 5*B*), but after sonication, there was a statistically significant loss of staining intensity as defined by integrated density (Fig. 5*A*). Staining for complement was also statistically significantly reduced after sonication-mediated removal of CPS as defined by integrated density (Fig. 5*A*).

When the sonicated cells were added to macrophages and the phagocytic index was measured, sonicated cells showed a statistically significant increase in phagocytosis in the presence of complement (Fig. 5*C*). In contrast, no change in phagocytic efficacy was observed with antibody-mediated opsonization with mAb 18B7 or when sonicated cells were non-opsonized (Fig. 5*C*).

### Models of capsule assembly

**Figure 6:**
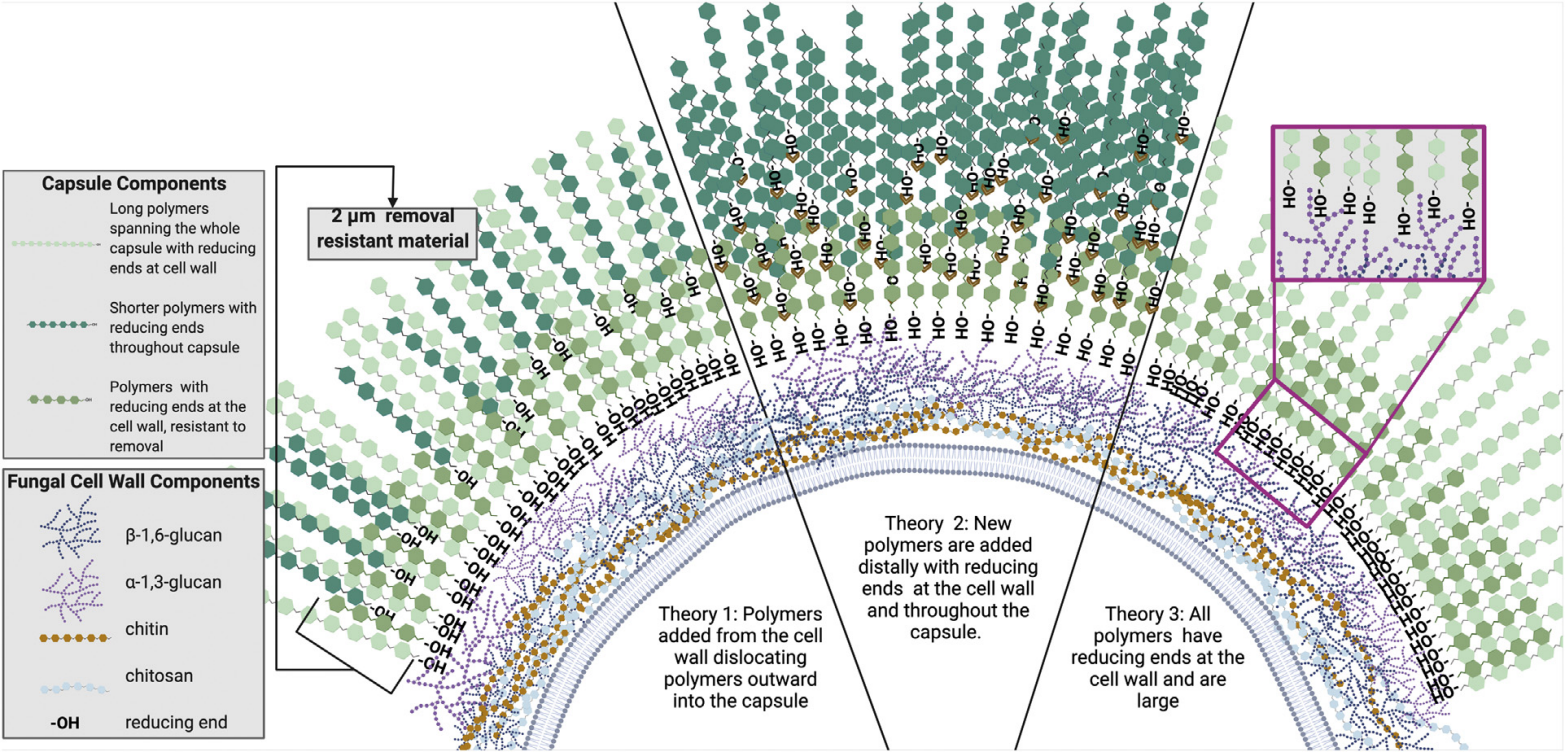
**Models of capsule rebuilding.** A diagram of three potential versions of capsule construction as informed by the data: Theory 1: Polymers are added at the cell wall dislocated outward. Theory 2: New polymers are added both at the cell wall and throughout the capsule. Theory 3: All polymers remain at the cell wall and some transverse the entire capsule. 2 μm of removal-resistant material is denoted. Figure created with BioRender (BioRender.com)

Three major theories of capsular assembly have been proposed. Theory 1 asserts that material is added at the inner portion of the capsule, thereby dislocating existing polymers and moving them outward from the cell (Fig. 6, Theory 1). This is supported by data with fluorescently labeled antibodies (37) and live imaging with antibodies (38). Theory 2 puts forth that capsule assembly occurs by incorporation of new material at the distal edge of the capsule (Fig. 6, Theory 2). This is supported by capsule regrowth after gamma irradiation (35). Theory 3 combines elements of Theories 1 and 2. In addition, a variation of theory 3 posits that the capsule is made of polymers which span the entire distance from the cell wall to the capsule edge (Fig. 6, Theory 3), at least for capsular enlargement. This is supported by the observation that polymer size and capsule radius are linearly correlated with polystyrene bead penetration (32) as well as recent experiments with the reducing end probe (34). Our observations showing reducing ends maintained at the cell wall–capsule interface in both the growing and mature capsule is consistent will all three theories. However, we also observe reducing ends throughout the capsule, but only when the reducing end probe is present during capsule assembly, suggesting that these are present during early assembly but blocked or not accessible in more mature capsules, a result that fits best with Theory 2. Finally, the size of polymers in CPS preparations do not show a linear correlation with the radius of capsule removed using these three methods, with the caveat that in this project we studied *de novo* capsule growth and not enlargement, as per the prior study, which used DMSO to remove CPS (32).

## Discussion

The capsule of *C. neoformans*, while critically important to fungal virulence, has eluded in-depth analysis due to both its hydrated nature (17) and its attachment to the cell wall. Structural insights have generally come from extrapolating the data derived from EPS, but work in 2008 concluded that EPS and CPS are distinct from one another physically and antigenically (12). In this work, we explored two physical methods, sonication and French press for capsule removal, and an enzymatic method, Glucanex digestion, and compared these to chemical removal with DMSO. Characterization of CPS isolated by these different methods reveals specific features and utility for each isolation type. While DMSO yields the greatest amount of CPS by ELISA (99 mg per 2 × 10^8^ cells), the NMR spectra do not contain an observable SRG peak set consistent with GXM motifs identified in EPS. This may be due to the large molecular weight of isolated polysaccharides resulting in line broadening, which abrogates the signal. Additionally, the DMSO method is time consuming, difficult, and kills the cells. Sonicated CPS, on the other hand, results in lower yields (46 mg per 2 × 10^8^ cells), but the observed SRG NMR peak set are consistent with GXM motifs identified in EPS. Further, the method is very facile with a quick turnaround, cells survive the treatment, and we observe capsule regrowth suggesting CPS can be isolated more than once using this method.

French press, modified Glucanex digestion, and lateral shearing, each resulted in the decapsulation of *C. neoformans*, but not into soluble polymers. India ink staining microscopy revealed that H99 cells exposed to relatively low pressures in the French press (∼500 psi) produced cells broken in half, but not fully stripped of capsular material. The cell wall was still present in these French press shells, as evidenced by Uvitex-2b staining. Pressures of 25,000 psi have been utilized to lyse cells (39) but far less pressure was necessary for *C. neoformans* capsular removal. After treatment with Glucanex and followed by cell disruption with vortexing, entire capsules without a cell body were observed, and we are calling these structures capsule ghosts. This phenomenon shows that it is possible to separate the capsule from the cryptococcal cell body, which may be attributable to the distinct cell wall architecture of cryptococcal cells. *C. neoformans* has less β-1,3-glucan, a target of Glucanex, than other yeasts, and it is localized to the exterior layer of the cell wall (40). Additionally, in the conditions used to produce Glucanex, *T. harzianum* also produces α-1,3-endo-glucanase which can directly cleave the α-1,3-glucan involved in GXM attachment to the cell wall (29, 41, 42). Repeated or excess pressure through lateral sheering can disrupt or break the cell body apart, resulting in off-center cell bodies, permeabilized capsules, and half cells like those observed during French press treatment. The shearing process appears to impact capsule–cell wall interactions as similar phenomena have been observed in cells with partial inhibition of chitin synthesis (43). Together, these data suggest that the removal of the capsule can occur in two ways, one which produces soluble polysaccharide particles, and another which allows capsular architecture to remain intact after separation from the cell body. The fact that the capsule can be separated from the cell body means that it is held together in ways that are not dependent on its contacts with the cell wall, suggesting the existence of a network of intermolecular linkages that hold the polysaccharide molecules together.

Of the four methods for capsule removal, sonication was associated with the highest cell survival and was therefore used for further immunogenic characterization. We observed that both antibody and complement bound to the partially stripped capsules, albeit at reduced amounts. This is consistent with previous work showing that the innermost region of the capsule has the lowest antibody binding capacity, while outer regions, removed by sonication, have higher predicted binding capabilities (5, 44). The patterns of antibody binding were unchanged by sonication, with the binding of mAb 2D10 remaining punctate before and after sonication, while mAb 2H1 exhibited sustained annular staining, consistent with previous findings (45). While the binding of mAbs 2H1 and 2D10 was decreased, the efficiency of antibody-mediated phagocytosis was not impacted by sonication. Even though complement deposition was decreased after sonication, sonicated H99 cells opsonized with complement had a greater percentage of phagocytic positive macrophages. Complement-mediated phagocytosis, unlike antibody-mediated phagocytosis (46), was previously shown to be inversely correlated with yeast cell size and is hypothesized to be impacted by deposition patterns (47). Together, these data indicate that the inner ∼2 μm of the capsule not removed by sonication differs from the outer region in antibody binding and complement-mediated phagocytosis.

Each of these methods yielded new insights into capsular architecture. Previous work using gamma irradiation–mediated CPS release showed capsule density lessens toward the periphery (35). Our work also indicates decreasing densities of ∼110 μg/μl at a distance of ∼1 μm from the cell, 20 μg/μl at 1.5 μm, 10 μg/μl at 2.3 μm, and <5 μg/μl at 3 μm (35). Using the same method of quantification, our isolated CPS average densities are 83 μg/μl for DMSO, 8 μg/μl for sonication, and 42 μg/μl for Glucanex digestion. While sonication yields a density in line with that estimated by Maxson *et. al.,* both DMSO and Glucanex digestion result in densities higher than those calculated based on CPS removal with gamma irradiation. Further study of the secondary, tertiary, and even quaternary structure of the capsule will be necessary to determine the complex interplay responsible for the different observed densities of the capsule.

The finding that *C. neoformans* can regrow capsule after removal is promising because it provides a new method for studying how the capsule is synthesized. Previous work to characterize capsular geography has involved the use of mAbs to GXM to assess capsule growth (46) and to determine how new polysaccharide polymers are added to the capsule (47). However, the binding of these antibodies can affect capsule structure through crosslinking effects (45) and antibody binding location could vary if epitope presentation changes during capsule growth. Here, we observe staining throughout the capsule as well as at the cell wall while we previously reported reducing end staining only at the cell wall–capsule interface (48). It is possible that reducing ends exist throughout the mature capsule and are not detected due to the relative high intensity of staining at the cell wall, that reducing end binding throughout the capsule results in more diffuse staining overall, or that time-dependent bleaching of the reducing end probe during growth experiments is occurring. Several different theories on capsule growth have been proposed. Some involve the intermingling of old and new polymers (48), while others suggest polymers extend from the cell wall to span the entire capsule (49). Here, we report that both *de novo* capsule growth and capsule regrowth after sonication contain reducing ends throughout the capsule. Dynamic light scattering analysis of the polymers in CPS isolates reveals size differences by method ranging from 0.28 μm (DMSO) at the smallest to 1.1 μm (Glucanex ghosts) at the largest, while the size of removed capsule total is ∼4 μm. These results add to, and confirm, a model of capsule assembly where smaller polymers are assembled distal to the ∼2 μm inner capsule layer to create the larger capsule.

In summary, we introduce three new methods to remove the *C. neoformans* capsule and use them to gain new insight into capsule architecture. The development of a method to strip the capsule while maintaining cell viability provides a novel system for studying how the capsule grows, which we use here to show that the young capsule has reducing ends that are inaccessible in the mature capsule, possibly as these are blocked during capsule maturation We also show that the method used to isolate CPS effects the polysaccharides, resulting in different amounts, particle sizes, and antigenic reactivity, a finding that should catalyze new studies of capsular PS composition and structure.

## Experimental procedures

### Yeast strains and culture

In capsule measurement, IF, French press, and phagocytosis experiments, H99 (serotype A), single expressing motif strain MU-1 (serotype A), 409 (serotype B), and 24,067 (serotype D) cells were subcultured into 6 ml Sabouraud liquid media for 2 days at 30 °C on a culture wheel. Cells were washed twice in PBS, and 3 ml of the culture was subcultured into 500 ml minimal media in a tissue culture flask at 30 °C with shaking (150 RPM). (15 mM D-glucose, 10 mM MgSO4• 7 H2O, 20.3 mM KH2PO4, 3 mM glycine, 10 mg/ml thiamine pH 5.5). For capsular material removal experiments, H99 (serotype A) was cultured in 5 ml YPD media for 2 days at 30 °C on a culture wheel. The cells were subsequently washed twice with PBS, and 500 μl was subcultured into 500 ml minimal media in a tissue culture flask at 30 °C with shaking (150 RPM). All cultures used grew in minimal media for 3 to 5 days. Strains were preserved in 15% glycerol and maintained at −80 °C.

### India ink/QCA

*C. neoformans* cells were mixed with india ink and imaged on an Olympus AX70 microscope using QImaging Retiga 1300 digital camera and the QCapture Suite V2.46 software (QImaging). Capsule measurements were produced using the exclusion zone produced with india ink and the Quantitative Capture Analysis program developed by the lab (31). Hundred yeast cells were measured for each strain and condition.

### Sonication

Cells cultured in minimal media for 3 to 5 days were washed three times with PBS and brought to a density of 1 × 10^7^ cells/ml. For capsule removal experiments, cells were brought to a density of 1 × 10^8^ cells/ml. After washing, 2 ml of cell suspension in PBS were sonicated on ice using a horn sonicator (Fisher Scientific Sonic Dismembrator F550 W/ultrasonic Convertor) on either setting 3 or 7 for 30 s (8 and 17 W, respectively). Sonicated cells used in phagocytic index experiments were washed postsonication to remove CPS.

### French press

H99 cells cultured in minimal media for 3 to 5 days were washed three times with PBS and brought to a density of 1 × 10^7^ cells/ml. Culture (10 ml) was run through the French press G-M High Pressure Standard Cell twice at a pressure of (500 psi). After passing cells through the French press, they were stained with 0.05% Uvitex 2B, and 1 μl of culture was combined with 7 μl india ink and imaged with Olympus AX70 microscope using QImaging Retiga 1300 digital camera and the QCapture Suite V2.46 software (QImaging).

### Glucanex digestion

Glucanex enzyme (Lysing Enzymes from *T. harzianum*) was prepared at a concentration of 50 mg/ml in spheroblasting buffer (1M sorbitol, 10 mM EDTA, and 100 mM sodium citrate). H99 cells cultured in minimal media for 3 to 5 days were washed three times with PBS and brought to a density of 1 × 10^8^ cells/ml. Cells (1 ml) were combined with 5 ml of Glucanex enzyme and incubated for 2.5 h at 30 °C while shaking. Cells were vortexed for 30 s on high or sonicated for 10 s on power 3 and then spun 7 min at 1200 RPM to isolated capsule ghosts.

### DMSO treatment

Washed H99 cells (500 μl) at a density of 1 × 10^8^ cells/ml were added to 15 ml of pure dimethyl sulfoxide (CH₃)₂SO. The cells and DMSO incubated for 30 min. At this time, the H99 cells were spun down and resuspended in 15 ml fresh DMSO. After 30 min, the H99 cells were spun down a second time. Total DMSO (30 ml) with capsular material was recovered. The remaining DMSO was removed from the sample by dialysis with at least 10 to 12 solvent changes.

### Capture ELISA

Microtiter plates were coated with anti-IgM Fc mAbs at a 1:1000 dilution in PBS at 50 ul per well. Plates were incubated at 37 °C for 1 h, then blocked with 200 ul blocking solution (1% BSA). After 1 h of blocking at 37 °C, plates were emptied by blotting over paper towels. Anti-GXM 2D10 IgG mAbs at 3 ug/ml, 50 ul per well were added. Plates were incubated at 4 °C overnight and washed three times with TBS with 0.1% Tween-20 before use. An EPS standard was prepared at a top concentration of 10 ug/ml, then serial diluted to make a total of eight points. CPS supernatants obtained from DMSO, sonication, and Glucanex treatment were diluted 500 times for the top concentration, then serial diluted to make a total of eight points. Serial dilutions were performed with blocking solutions. EPS standard (50 ul) and samples at different dilutions were added, and blocking solution was used as a negative control. Plates were incubated at 37 °C for 1 h and then washed three times. Anti-GXM 18B7 IgM mAbs at 5 ug/ml, 50 ul/well were added, plates were incubated for 1 h, and then washed three times. Alkaline phosphatase–labeled anti-IgG Abs at 1 ug/ml, 50 ul/well were added, plates were incubated for 1 h, and washed five times. Finally, 1 mg/ml, 50 ul/well alkaline phosphatase substrate in substrate buffer were added. Plates were developed at RT and read at A405 until the top concentration standard absorbances reached just over 1. EPS A405 absorbances were plotted against concentrations and then fit with a linear model. This linear relationship was used to estimate the concentrations of CPS samples with respect to their A405 absorbances.

### PSA assay

This assay was modified from Masuko *et al*., 2005 (49). To start, 100 μl of 1M mannose was 1:2 serially diluted into 50 μl of sterile water in the first three columns of a 96-well plate. Fifty microliters of each sample were put into three wells undiluted. A volume of 50 μl of sterile water was placed in three wells to determine background. One hundred microliters of concentrated sulfuric acid were placed in each well. Thirty microliters of a 5% phenol solution were pipetted into each well. The 96-well plate was then incubated at 37 °C for about 10 min. Plate was read at read at 490 nm.

### Statistical correlations of capsule degradation measurements

For each experimental capsule removal experiment (CPS removal or antibody-mediated capsule degradation), polysaccharide concentration in the supernatant was measured by capture ELISA, PSA, and dry weight measurements. The total polysaccharide amount was then calculated to correct for differences in total volume. The corresponding *C. neoformans* cells were also analyzed after CPS removal or antibody treatment to measure the average capsule diameter. These measured values were then plotted as a scatter plot with respect to two variables at a time, and the correlation coefficient by the Spearman nonparametric correlation test was calculated in GraphPad Prism 9.2.0.

### Immunofluorescence

To visualize the antibody staining, 5 × 10^6^ total cells were resuspended in 1 ml blocking solution (prepared fresh, 1% BSA in 100 ml PBS) with 10 μg/ml of either of the primary mAbs that detect different epitopes within the PS capsule of *C. neoformans*. This study compared one IgM mAb, 2D10, and one IgG mAb, 2H1, antibodies. Complement binding changes were assessed by incubated cells in blocking buffer and 20% complement (guinea pig complement, MilliPore). H99 cells with either mAb or complement were placed on a shaker for 1 h at 37 °C. After primary opsonization, cells were spun down and washed with PBS then resuspended in 1 ml blocking solution with either 1:500 Goat anti-Mouse IgM Heavy Chain Secondary Antibody conjugated to AlexaFluor 488 (Invitrogen), 1:500 Rabbit anti Mouse IgG (H + L) Secondary Antibody, Alexa Fluor 594, Invitrogen, or 1:50 goat anti-Complement C3 polyclonal—FITC (Invitrogen). H99 cells with either fluorescently conjugated anti-IgM mAb, anti-IgG mAb, or fluorescently conjugated anti-complement mAb were placed on a shaker for 1 h at 37 °C. Post-incubation cells were washed once in PBS and imaged with Pro-Long Gold mounting solution (Molecular Probes). FITC channel excitation/emission was 498 and 516 nm respectively and TRITC excitation/emission was 540 and 580 nm respectively. Exposures of 2H1, 2D10, and complement binding were 100 ms, 400 ms, and 700 ms respectively. The same exposure was applied to both control and sonicated images. Images were collected with an Olympus AX70 microscope, photographed with a QImaging Retiga 1300 digital camera using the QCapture Suite V2.46 software (QImaging). Quantification of cells was achieved with ImageJ from Fiji (NIH) by measuring integrated density around each cell and subtracting background intensity from each measurement. No adjustment of brightness or contrast was performed for Figure 3. Color filters were assigned with ImageJ from Fiji (NIH). All images in Figure 5 were simultaneously brightened to the same degree in photoshop to view Uvitex2B/india ink contrast. Control and sonicated cells were measured with an area of the same size and the subtracted background was additionally scaled to this size.

### Phagocytic index

Bone marrow–derived macrophages (BMDMs) were obtained from 6-week-old C57BL/6 female mice. After 5 to 7 days of differentiation, the cells were seeded 5 × 10^4^ cells per Mat-Tek dish and allowed to settle for 30 min. After 30 min, the dish was supplemented with 2 ml media. BMDMs were activated overnight with 0.5 μg/ml lipopolysaccharide (MilliporeSigma) and 10 ng/ml IFN-γ (Roche) for M1 polarization. BMDMs were maintained at 37 °C 9.5% CO_2_. *C. neoformans* were prepared and sonicated as described above. 1.5 × 10^5^ H99 were added for an MOI of 3. Opsonized cells were prepared with 10 μg/ml 18B7 or 20% complement (Fisher Scientific #642831) and placed on the slide portion of the dish. After a 2-h infection at 37 °C 9.5% CO_2_, BMDM cells were washed 4× with media to remove extracellular *C. neoformans* cells. Cultures were visualized on a Zeiss Axiovert 200M inverted microscope with a 10× phase objective in an enclosed chamber at 9.5% CO2 and 37 °C. Hundred BMDMs of each condition were counted and then scored for the presence of *C. neoformans* cells.

### Capsule regrowth after sonication and Glucanex digestion

H99 cells were cultured, brought to a final concentration of 10^7^ cells/ml, and 2 ml cells were sonicated and treated with Glucanex followed by sonication as described above. CPS supernatants were obtained after centrifugation, and treated cells were resuspended in 20 ml minimal media and incubated with shaking at 30 ^°^C for capsule regrowth. India ink slides were imaged and analyzed by QCA at several timepoints; 1 ml cells were aliquoted out and concentrated into 10 ul PBS for imaging. The sonicated cells were re-sonicated after approximately 48 h of regrowth; half of the treated cells were directly resuspended in 20 ml minimal media for a second regrowth, and the other half were stained by a reducing end probe by adding 10ul HA-488 at a concentration of 10 mg/ml in DMSO to 20 ml minimal media into which the cells were resuspended. India ink slides were imaged under white light to track capsule regrowth. To measure reducing end localization, cells were images on a Zeiss AxioImager M2 upright microscope using GFP and brightfield. Both sonicated cells regrown with and without HA-488 were washed twice in PBS and imaged at an exposure of 474 ms in the GFP channel. To confirm staining was not due to sonication alone, follow-up experiments were performed comparing cells grown in minimal media for 3 days at 30 °C either with 1 μl of HA-488 probe per mL during growth or added after growth for 1 h. Cells were washed twice in PBS and imaged at an exposure of 600 ms in the GFP channel. To estimate localization, plot profiles of >30 cells were produced and normalized to their peak intensity to compare the location of the probe throughout the sample in samples with differing stain intensities at the cell wall. Plot profiles were created in Fiji (NIH). To ensure the entire capsule was included in the plot profile, a line was drawn across the diameter of the cell in the india ink brightfield image, and the plot profile of the GFP image was measured.

### Solution NMR

1D ^1^H NMR data were collected on a Bruker Avance II (600 MHz), equipped with a triple resonance, TCI cryogenic probe, and z-axis pulsed field gradients. Spectra were collected at 60 °C, with 128 scans and a free induction decay size of 84,336 points. Standard Bruker pulse sequences were used to collect the 1D data (p3919gp and zggpw5). Data were processed in Topspin (Bruker version 3.5) by truncating the free induction decay to 8192 points using a squared cosine bell window function and zero filling to 65,536 points.

Lyophilized samples were solubilized in deuterated water to a concentration of 500 mg/ml. Native samples were diluted by adding 300 μl of D_2_O to 200 μl of sample. All NMR samples contained DSS-d_6_ for chemical shift calibration and peak intensity comparisons.

Spectra were deconvoluted using the MNova program (through NMRbox) line fitting algorithm with a Lorentzian-Gaussian shape type and either 100 or 500 iterations to identify the SRG region peak sets.

### Dynamic light scattering

Measurement of particle size by dynamic light scattering was performed with a Zeta Potential Analyzer instrument (Brookhaven Instruments). The particle sizes in the suspension were measured for all samples, and data are expressed as the average of 10 runs of 1-min data collection each. Each PS preparation (100 μl) was added to an UVette Cuvette (Eppendorf) at room temperature. The multimodal size distributions of the particles were obtained by a non-negatively constrained least squares algorithm based on the intensity of light scattered by each particle. The multimodal size distributions of particles from each sample were graphed for comparison.

## Data availability

All data are contained within the manuscript. Strains used in this study are available by request of the corresponding author.

## Supporting information

This article contains supporting information.

## Conflict of interest

The authors have no conflicts of interest to report.

## Supporting information

Supplemental Figure 1 **Cell body size varies by *C. neoformans* strain.** Four strains were evaluated for cell body size using india ink images and the QCA assay. The cell bodies of single motif expressing strains 24067, 409, and Mu-1 are smaller than those of mixed motif expressing strain H99. n =100 cells were analyzed for each strain. QCA, quantitative capsule analysis.

Supplemental Figure 2 **DLS analysis of the size of particles released by CPS isolation methods.***A*, the multimodal size distributions of three biological replicates of H99 exposed to sonication and DMSO treatment. Full particle diameter range for each biological replicate shows variability in size between both replicates and method of CPS isolation. On average, particle sizes are similar between DMSO and sonication treatments. *B*, multimodal size distribution of one biological replicate of Serotype A strain Mu-1 exposed to sonication and DMSO treatment. *C*, multimodal size distribution of one biological replicate of serotype B strain 409 exposed to sonication and DMSO treatment. Full particle diameter range for each treatment shows similar variability to H99 samples with the same overall trend of similar average particle size being produced in both sonication and DMSO treated cells. DMSO, dimethyl sulfoxide; CPS, capsular polysaccharide.

Supplemental Figure 3 **NMR analysis of CTAB–EPS, sonicated CPS, and DMSO extracted CPS of two single-motif expressing strains.** The SRG region of 1D NMR spectra were examined for GXM signature peaks in all three samples. A Lorentzian-Gaussian deconvolution was applied to the spectra to identify GXM motif peak sets. (*top*) CTAB–EPS served as the positive control and contained the signature peak set for each strain’s representative GXM motif. *(middle)* Sonication CPS samples show the signature GXM peak sets only after 500 iterations of deconvolution (standard is 100). *C*, DMSO CPS spectra for both strains were inconclusive due to high signal-to-noise ratio and did not contain the signature peak set representative of the respective GXM motifs. DMSO, dimethyl sulfoxide; CPS, capsular polysaccharide; CTAB, cetyl trimethylammonium bromide; EPS, exopolysaccharide; GXM, glucuronoxylomannan; SRG, structural reporter group.

Supplemental Figure 4 **Impact of lateral shear, and Glucanex on capsular architecture.***A*, immunofluorescence microscopy images of capsule ghosts produced by Glucanex showing no cellular components (as stained by DAPI), but GXM as stained by 18B7-488. *B*, india ink stained Glucanex-derived capsule ghosts show blebbing of the capsule and loss of cell walls. *C*, lateral shear generates capsule ghosts. Counterstained micrographs of encapsulated H99 *C. neformans* cells before (i) and after applying lateral shear pressure showing cell body and capsule dislocation (ii-iii) with scale bar representing 5 μm. Lateral sheared cells stained with Ubitex2B show india ink penetration of the capsule after treatment (lower panel). Scale bars represent 100 μm. All images adjusted for brightness and contrast in the same manner. GXM, glucuronoxylomannan.

Supplemental Figure 5 **Confirmation of capsular probe detection.** Plot profiles of 17 unstained H99 cells compared to 17 sonicated cells stained with HA-488. The 17 stained plot profiles were also used in the production figure 4. Both were imaged at 474 ms in the GFP filter to confirm capsule probe detection was not due to capsular autofluorescence in the GFP filter. Graph shows averaged unnormalized plot profiles with no cell body removal.
