# Supplementary figures and images for: Cryptococcus neoformans capsule regrowth experiments reveal dynamics of enlargement and architecture
Source: J Biol Chem. 2022 Feb 24;298(4):101769. doi: 10.1016/j.jbc.2022.101769 (PMC8942833; doi:10.1016/j.jbc.2022.101769)

# Cell Body Sizes

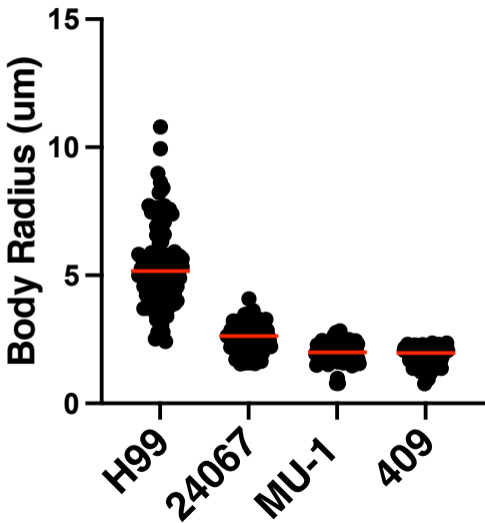

Supplement: Supplemental Figure 1 — Cell body size varies by C. neoformans strain. Four strains were evaluated for cell body size using india ink images and the QCA assay. The cell bodies of single motif expressing strains 24067, 409, and Mu-1 are smaller than those of mixed motif expressing strain H99. n =100 cells were analyzed for each strain. QCA, quantitative capsule analysis. [file mmc1.pdf]

**A****H99 Sonicated**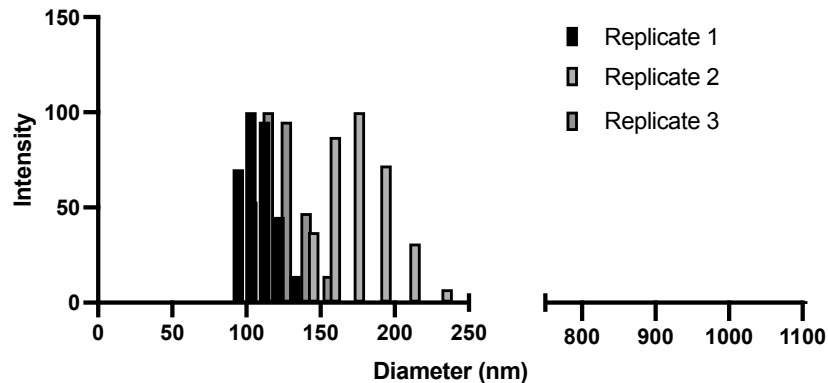**B****MU-1 Sonicated**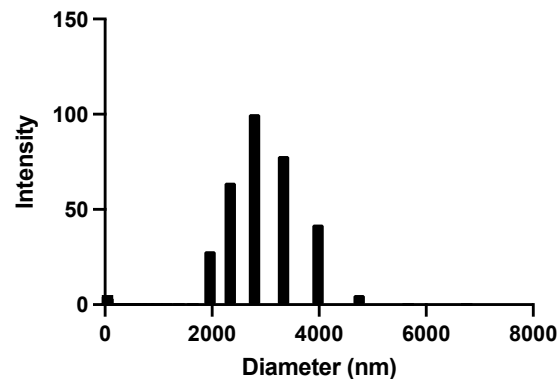**C****409 Sonicated**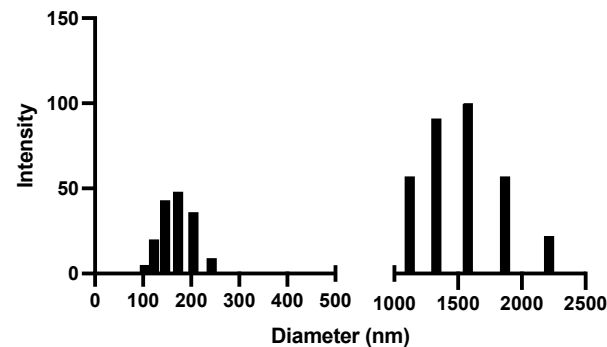**H99 DMSO**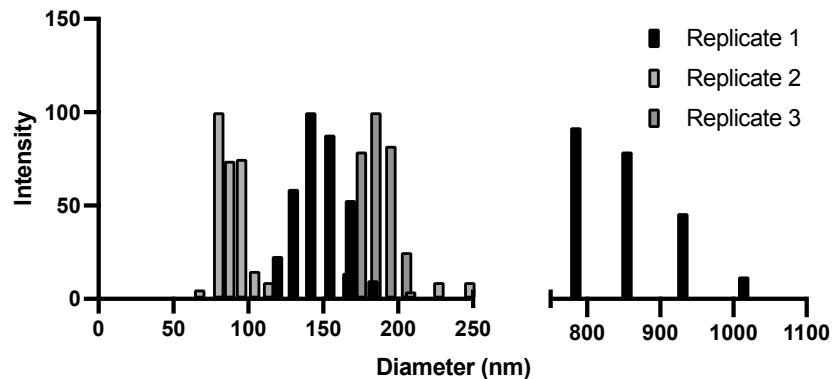**MU-1 DMSO**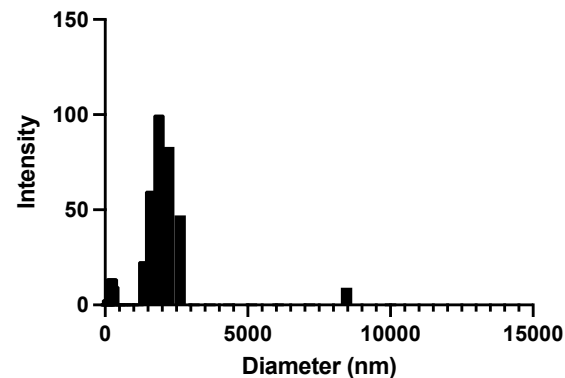**409 DMSO**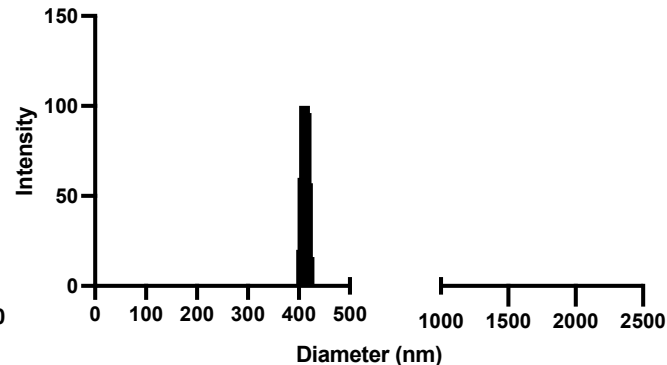

Supplement: Supplemental Figure 2 — DLS analysis of the size of particles released by CPS isolation methods.A, the multimodal size distributions of three biological replicates of H99 exposed to sonication and DMSO treatment. Full particle diameter range for each biological replicate shows variability in size between both replicates and method of CPS isolation. On average, particle sizes are similar between DMSO and sonication treatments. B, multimodal size distribution of one biological replicate of Serotype A strain Mu-1 exposed to sonication and DMSO treatment. C, multimodal size distribution of one biological replicate of serotype B strain 409 exposed to sonication and DMSO treatment. Full particle diameter range for each treatment shows similar variability to H99 samples with the same overall trend of similar average particle size being produced in both sonication and DMSO treated cells. DMSO, dimethyl sulfoxide; CPS, capsular polysaccharide. [file mmc2.pdf]

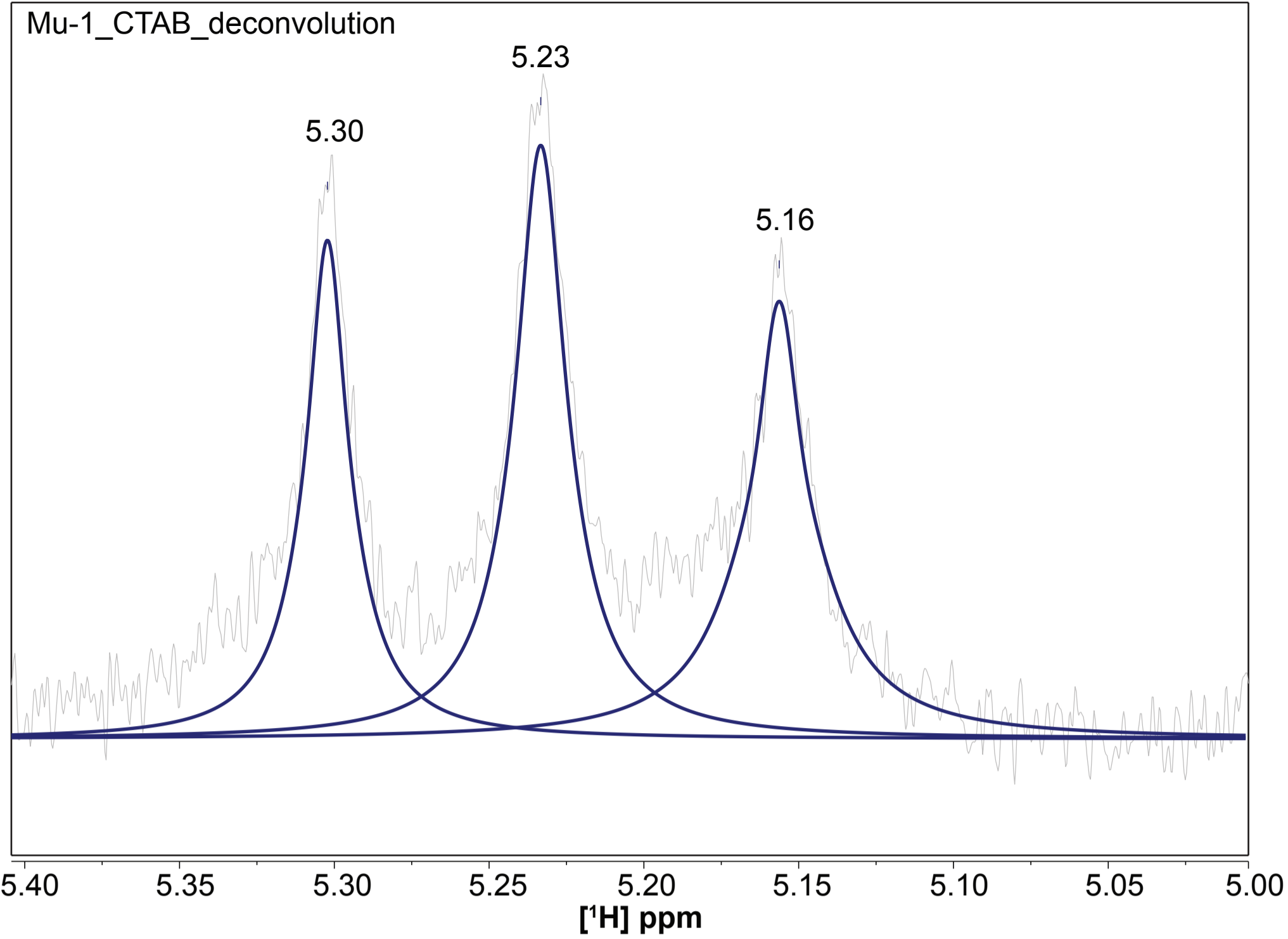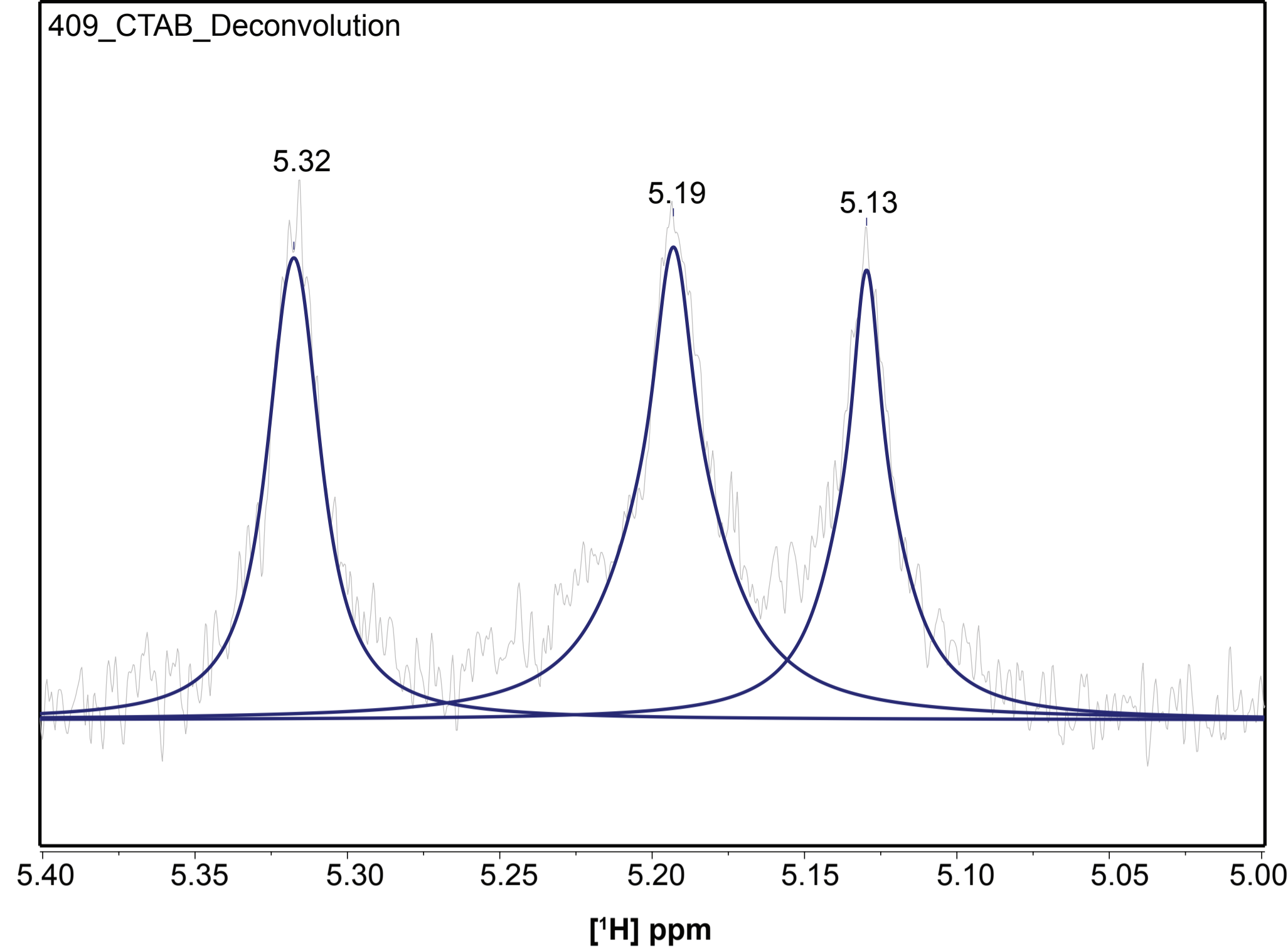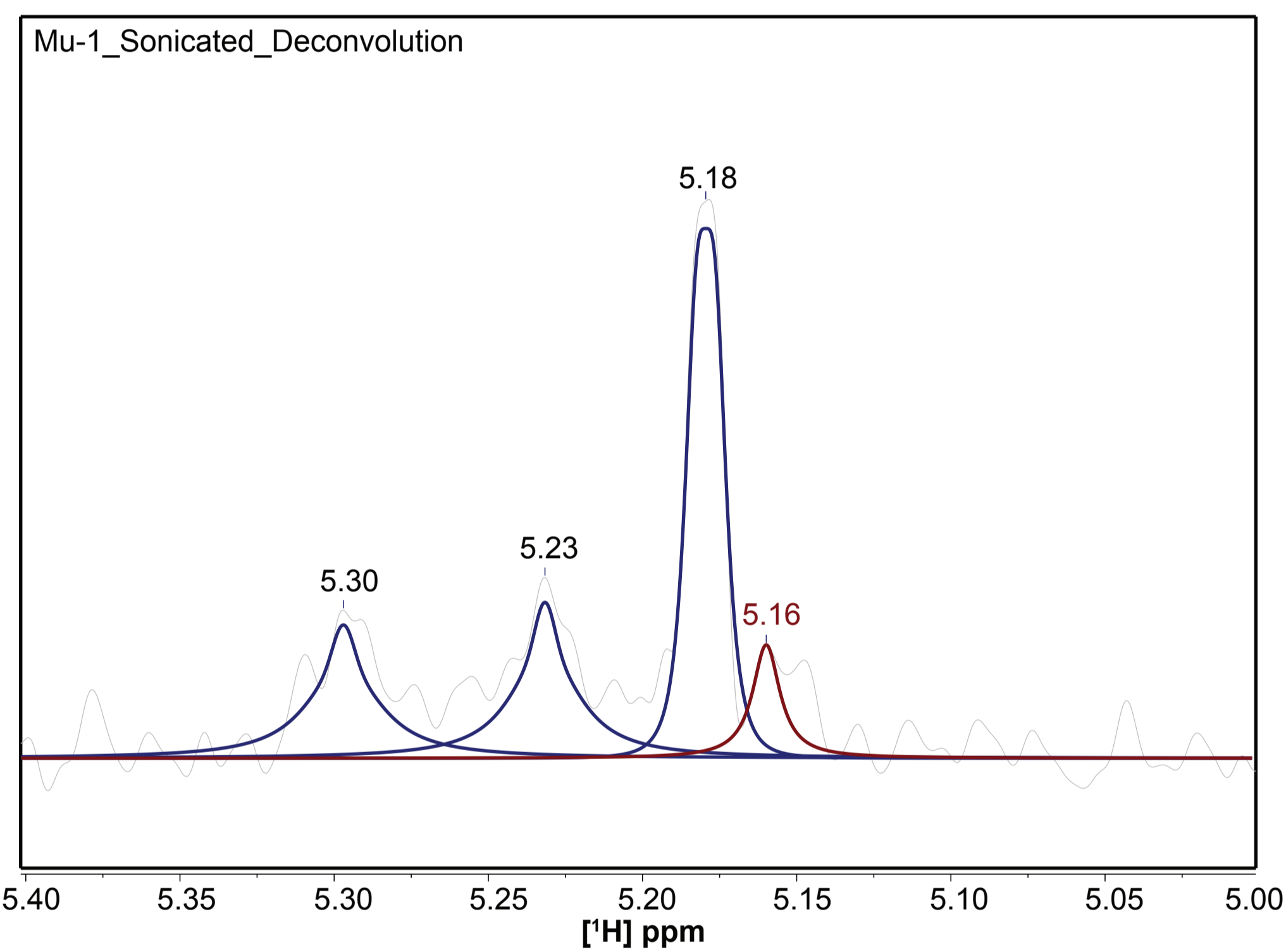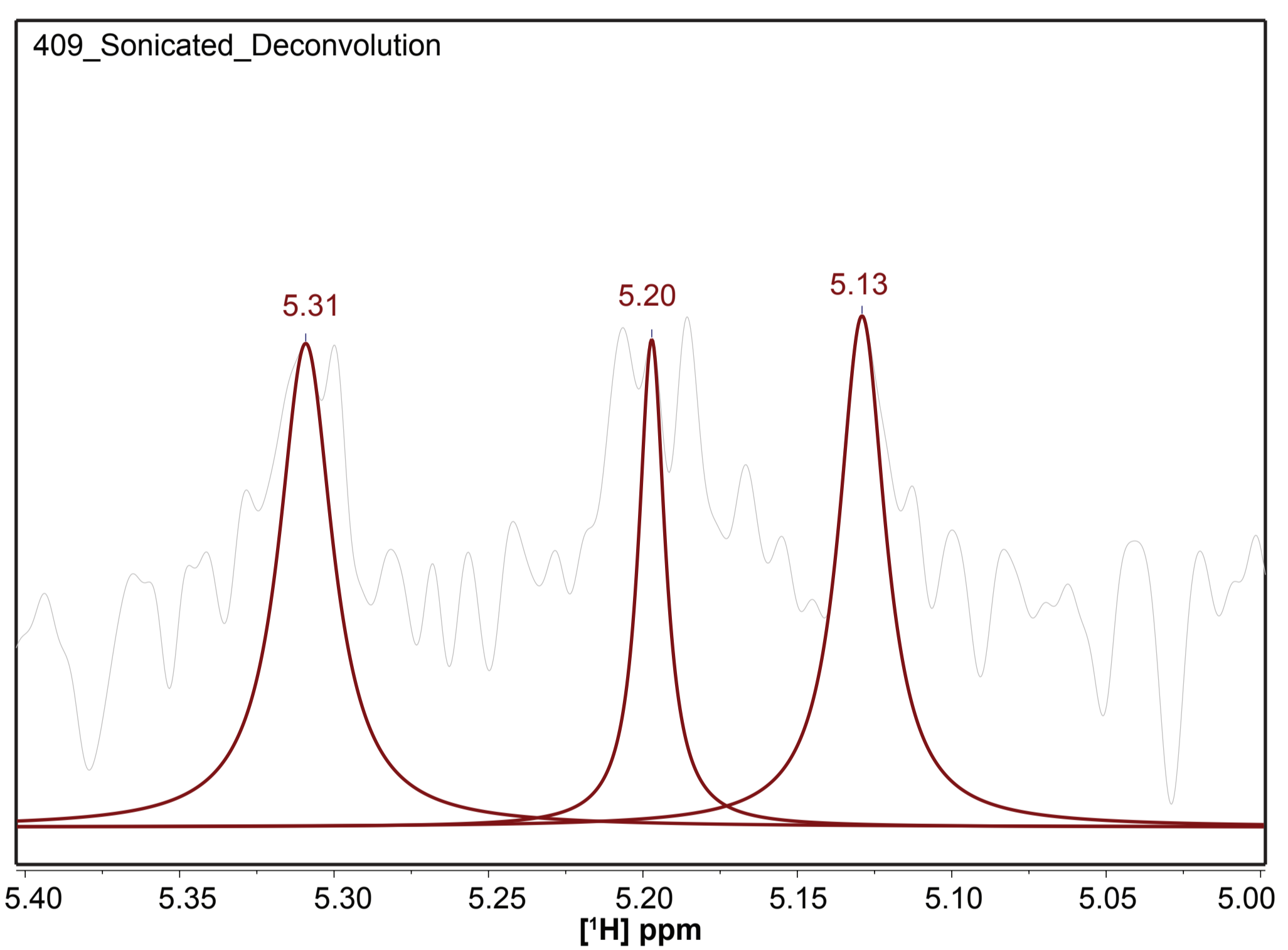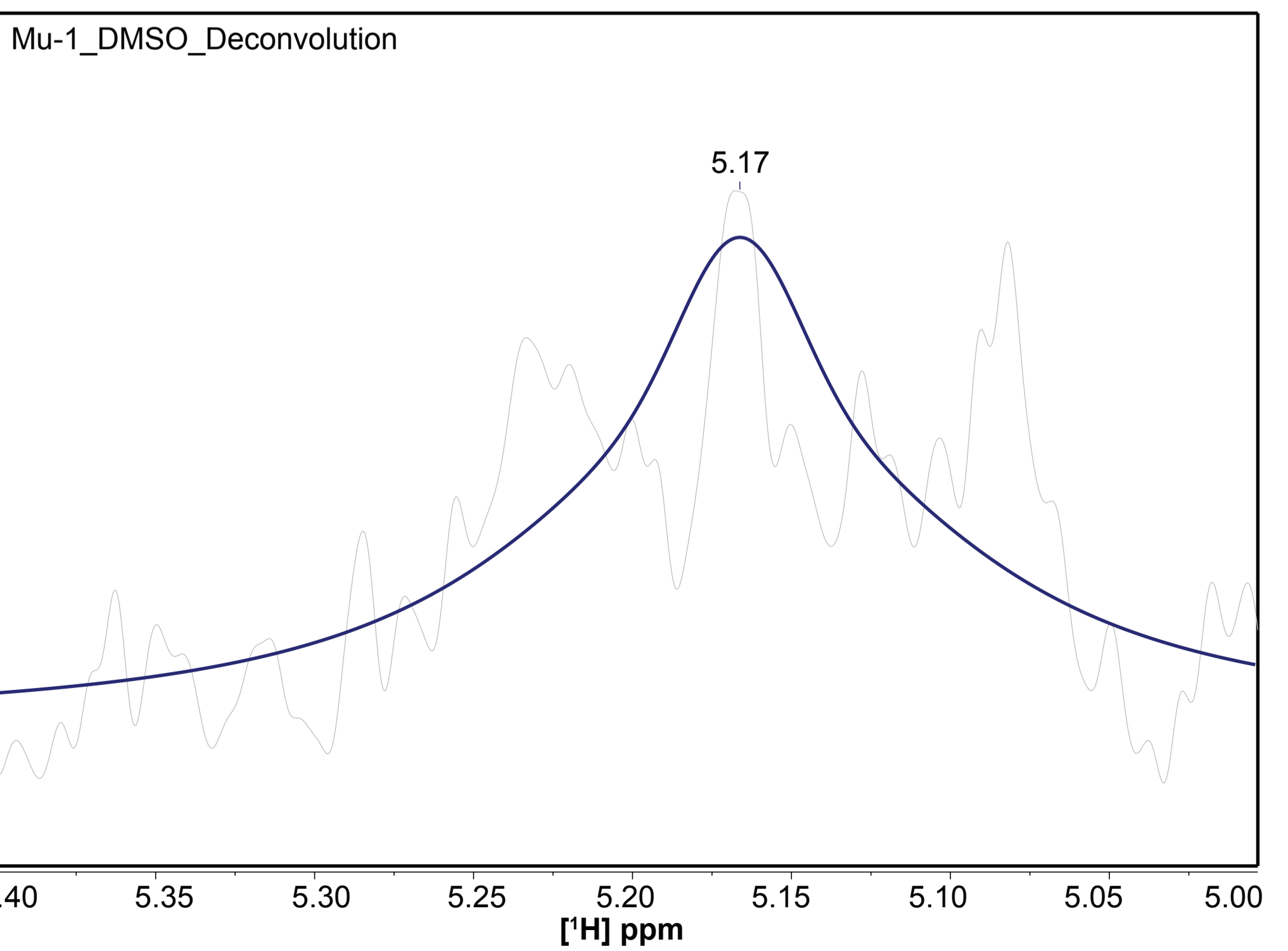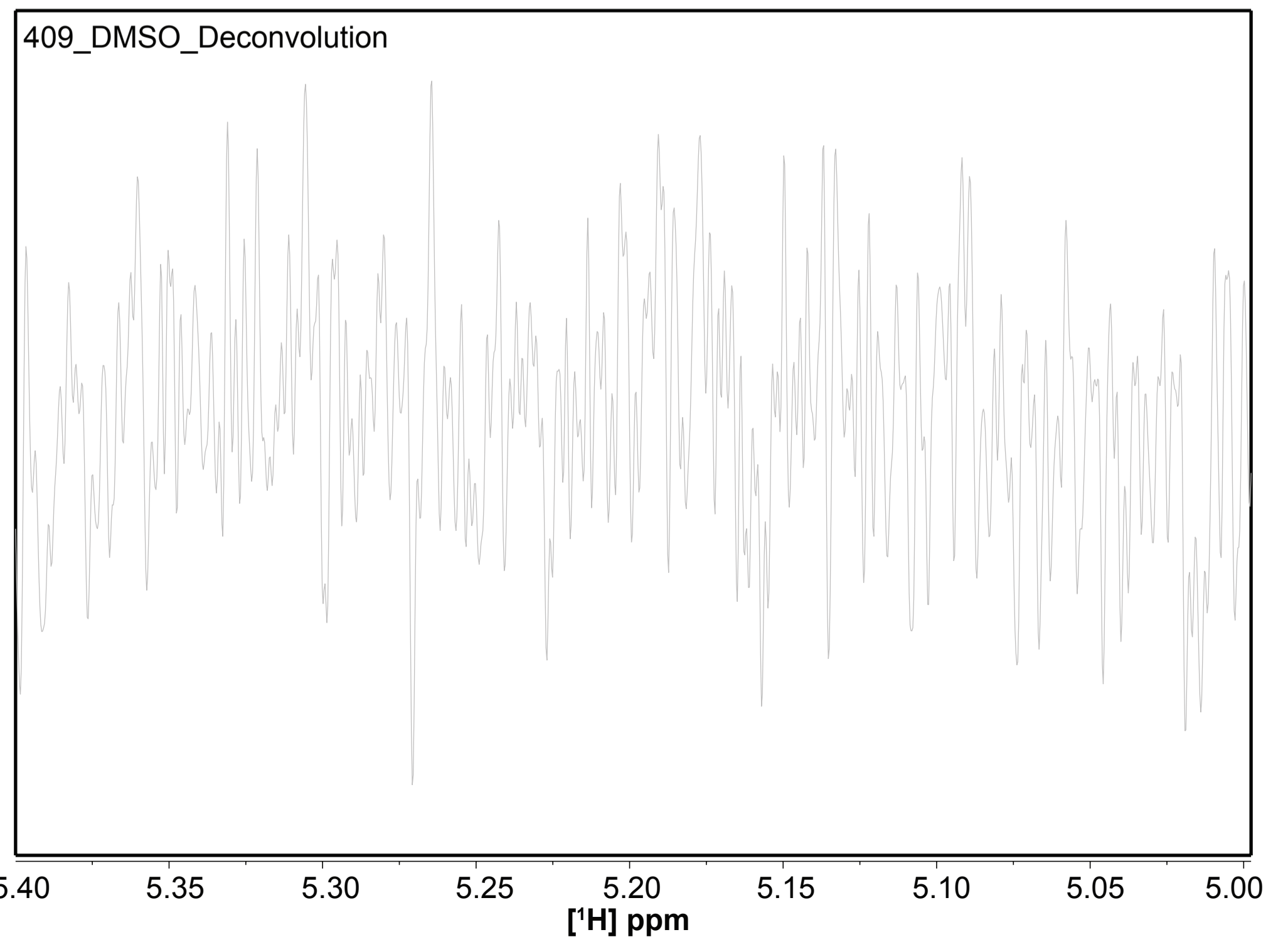

Supplement: Supplemental Figure 3 — NMR analysis of CTAB–EPS, sonicated CPS, and DMSO extracted CPS of two single-motif expressing strains. The SRG region of 1D NMR spectra were examined for GXM signature peaks in all three samples. A Lorentzian-Gaussian deconvolution was applied to the spectra to identify GXM motif peak sets. (top) CTAB–EPS served as the positive control and contained the signature peak set for each strain’s representative GXM motif. (middle) Sonication CPS samples show the signature GXM peak sets only after 500 iterations of deconvolution (standard is 100). C, DMSO CPS spectra for both strains were inconclusive due to high signal-to-noise ratio and did not contain the signature peak set representative of the respective GXM motifs. DMSO, dimethyl sulfoxide; CPS, capsular polysaccharide; CTAB, cetyl trimethylammonium bromide; EPS, exopolysaccharide; GXM, glucuronoxylomannan; SRG, structural reporter group. [file mmc3.pdf]

**A**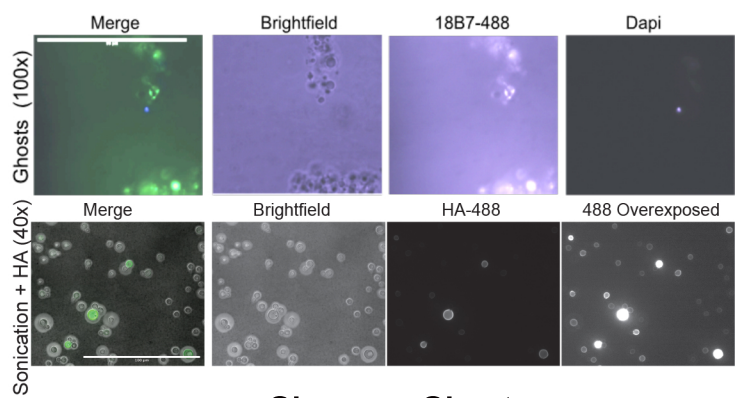**B**

### Glucanex Ghosts

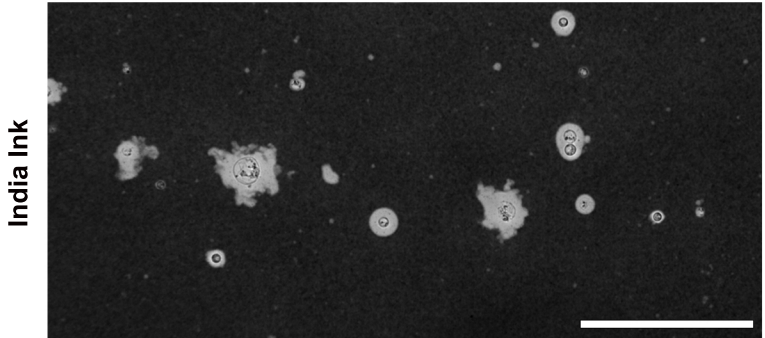**C**

### Lateral Shear

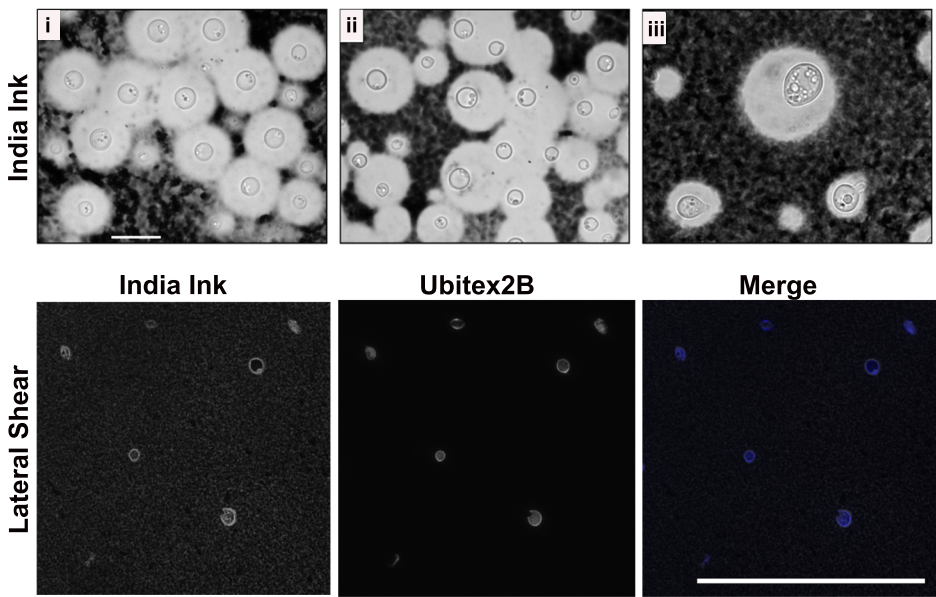

Supplement: Supplemental Figure 4 — Impact of lateral shear, and Glucanex on capsular architecture.A, immunofluorescence microscopy images of capsule ghosts produced by Glucanex showing no cellular components (as stained by DAPI), but GXM as stained by 18B7-488. B, india ink stained Glucanex-derived capsule ghosts show blebbing of the capsule and loss of cell walls. C, lateral shear generates capsule ghosts. Counterstained micrographs of encapsulated H99 C. neformans cells before (i) and after applying lateral shear pressure showing cell body and capsule dislocation (ii-iii) with scale bar representing 5 μm. Lateral sheared cells stained with Ubitex2B show india ink penetration of the capsule after treatment (lower panel). Scale bars represent 100 μm. All images adjusted for brightness and contrast in the same manner. GXM, glucuronoxylomannan. [file mmc4.pdf]

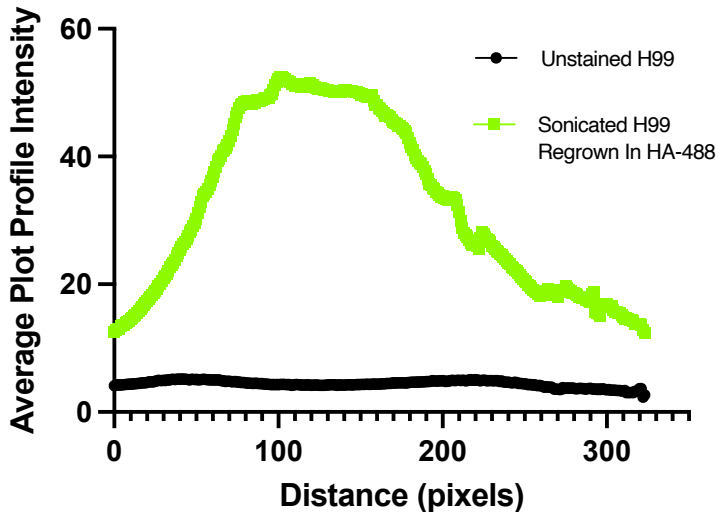

Supplement: Supplemental Figure 5 — Confirmation of capsular probe detection. Plot profiles of 17 unstained H99 cells compared to 17 sonicated cells stained with HA-488. The 17 stained plot profiles were also used in the production figure 4. Both were imaged at 474 ms in the GFP filter to confirm capsule probe detection was not due to capsular autofluorescence in the GFP filter. Graph shows averaged unnormalized plot profiles with no cell body removal. [file mmc5.pdf]
